# Investigation of Netrin-1 Levels in Maternal and Umbilical Cord Serum in Preeclampsia and Their Relationship With Vitamin D, Vitamin B12, and Folic Acid

**DOI:** 10.7759/cureus.102489

**Published:** 2026-01-28

**Authors:** Gulsah Cetindari Demirci, Soner Gök, Berfin Gök, Esin Avci

**Affiliations:** 1 Gynecology and Obstetrics Clinic, Kemer State Hospital, Antalya, TUR; 2 Gynecology and Obstetrics, Pamukkale University, Denizli, TUR; 3 Gynecology and Obstetrics, Denizli State Hospital, Denizli, TUR; 4 Medical Biochemistry, Pamukkale University Hospital, Denizli, TUR

**Keywords:** folic acid, netrin-1, preeclampsia, vitamin b12, vitamin d

## Abstract

Background

The purpose of this study was to compare maternal blood and umbilical cord Netrin-1 levels at delivery in severe preeclampsia (PE) patients to a control group, investigate their relationship with clinical parameters, and look into the potential link between maternal vitamin D, vitamin B12, and folic acid levels.

Methods

This is a case-control observational study that included 22 pregnant women with severe PE and a control group of 22 healthy pregnant women of the same gestational age. We measured Netrin-1 levels in serum samples collected from maternal blood and umbilical cords during cesarean section in both groups. Vitamin D, B12, and folic acid levels in maternal blood were also measured in both groups. An enzyme-linked immunosorbent assay was used to measure Netrin-1 levels.

Results

The maternal Netrin-1 levels were significantly higher in the PE group compared to the control group (p = 0.006). We found a significant positive correlation between maternal Netrin-1 levels and proteinuria in the PE group (p = 0.001). The control group, on the other hand, did not exhibit this relationship.

Conclusion

We discovered that PE cases had higher levels of maternal Netrin-1, and that maternal Netrin-1 levels and proteinuria were significantly positively correlated. Nevertheless, we did not find any correlation or increase in umbilical cord Netrin-1 levels. We believe that the primary reason for these changes is to protect the fetus from microvascular and endothelial injury.

## Introduction

Proteinuria and hypertension are the hallmarks of preeclampsia (PE), a pregnancy-related condition that usually manifests around 20 weeks of gestation. A significant contributor to maternal and perinatal morbidity and mortality worldwide, this condition affects 2-8% of pregnancies [1]. Medical professionals believe that PE is caused by placental insufficiency caused by inadequate remodeling of the maternal vascular bed, though the exact mechanism is unknown. Angiogenetic imbalance contributes significantly to PE pathophysiology [2]. Patients with PE develop hypertension, endothelial dysfunction, and proteinuria when anti-angiogenic factors interfere with angiogenic factor action [3]. Proteinuria is no longer considered the gold standard for diagnosing PE, despite being a visible symptom. Pulmonary edema, liver failure, hypertension, and recently developed renal failure, thrombocytopenia (platelet count <100,000/microliter), and abnormalities in vision and cognition are all included in PE [1]. It has the potential to significantly increase maternal mortality and morbidity over the short, medium, and long term. Furthermore, it may have serious consequences for the fetus, such as premature delivery, intrauterine growth restriction, or prenatal death [4,5].

Netrins can have pro- or anti-angiogenic effects depending on the receptor and how it is expressed [6]. Netrins play an important role in angiogenic processes, according to recent research. They are essential for the formation of both the placenta's vascular system and the placenta itself [7]. According to recent research, the laminin-like protein Netrin-1, which is required for neurovascular growth, may also contribute to PE's development. Netrin-1 has been shown to be an important regulator of placental development and vascular remodeling, with anti-apoptotic, anti-inflammatory, and angiogenic properties [8].

PE has been associated in certain studies with vitamin D deficiency [9]. Vitamin D has been shown to protect against PE by regulating blood pressure, immunomodulating, anti-inflammatory, and antioxidant processes [9]. The placenta and other organs express vitamin D receptors (VDR), which are believed to be the mechanism by which the active form of vitamin D, 1,25(OH)2D, functions [10]. While some studies have linked vitamin D deficiency to a higher risk of PE [9], others have found conflicting results [11-13]. While some of these studies discovered no link between low maternal vitamin D levels and the development of PE, others did [9,11].

Recently, some researchers investigated how maternal vitamin B12, homocysteine, and folate levels influence the development of PE [12,13]. There is increasing evidence that hyperhomocysteinemia may contribute to endothelial dysfunction in PE, which is caused by oxidative stress [14]. Homocysteine is an intermediate amino acid that is converted to cysteine or methionine. For homocysteine, including methionine synthase, to function, folate must be a cosubstrate and vitamin B12 must be a cofactor [13]. Increased circulating homocysteine levels have been linked to low folate and vitamin B12 levels, which raises the risk of PE [14].

This study's objectives were to measure Netrin-1 levels in the maternal and fetal cord serum of preeclamptic pregnant women, examine how these levels relate to clinical parameters, and look into possible connections between levels of folic acid, vitamin D, and vitamin B12. We believe that these findings can help to clarify Netrin-1's role in PE pathogenesis and lead to the development of new treatment strategies.

## Materials and methods

This case-control, observational study was conducted at Pamukkale University Hospital's Obstetrics and Gynecology Clinic from June 2021 to January 2022, with the Pamukkale University Local Ethics Committee's approval (E-60116787-020-61293, dated 08/06/2021). This study was supported by the Pamukkale University Research Fund (2021TIPF023).

Study design and patients

The study included 113 pregnant women aged 18 to 40 who applied to the obstetrics and gynecology department, agreed to participate in our study, and had a cesarean section between weeks 34 and 36 of their pregnancy. The study excluded 16 pregnant women with systemic diseases, 14 pregnant women with gestational diabetes mellitus (GDM), three pregnant women with postpartum hypertension, 18 pregnant women with premature membrane rupture, five pregnant women whose urine microscopy revealed bacteria, and three pregnant women who declined to take part. Two cases from the control group and eight cases from the PE group were excluded from the study because their serum analysis results were significantly higher or lower than the mean, which was incompatible with reality. The PE group consisted of 22 pregnant women who fit the severe PE diagnostic criteria. Severe PE was the reason for delivery in the PE group, whereas premature birth (spontaneous birth with intact membranes) at 34 or 36 weeks of gestation was the reason for delivery in the control group. The control group included 22 pregnant women. The current births in both groups were also cesarean sections because all of the cases had previously had one. Due to their gestational age of 34 weeks or more, neither pregnant woman in either group received prenatal steroids.

Exclusion criteria included pregnancy, eclampsia, known serious chronic conditions, preexisting cancer, urinary tract infections, preexisting chronic renal disease that affected urine output, and a poor obstetric history that might affect the cardiovascular system during pregnancy.

During their hospital stay and the six-week postpartum period, blood pressure was closely monitored for all study participants, including those in the PE and control groups.

First-trimester ultrasonography of the crown-rump length was used to confirm the gestational ages, which were determined using the first day of the most recent menstrual cycle.

The diagnostic criteria for severe PE were developed by the American College of Obstetricians and Gynecologists [15]. These standards include the following: (1) thrombocytopenia (platelet count less than 100 x 109/L); (2) systolic blood pressure of 160 mmHg or higher or diastolic blood pressure of 110 mmHg or higher on two occasions at least four hours apart (unless antihypertensive therapy is started before this time); (3) impaired liver function not explained by alternative diagnoses and as indicated by abnormally elevated blood concentrations of liver enzymes (to more than twice the upper quadrant or epigastric pain that is severe and unresponsive to medication; (4) pulmonary edema; (5) renal insufficiency (serum creatinine concentration greater than 1.1 mg/dl or a doubling of the serum creatinine concentration without another renal illness); (6) a new-onset headache that doesn't go away with medicine and can't be explained by other conditions; and (7) visual problems. 

Each participant's height and weight were measured prior to delivery, and their BMI values (kg/m2) were computed.

Sample collection and measurement of serum levels of vitamin D, folic acid, vitamin B12, and Netrin-1

Urine samples were collected from all participants over a 24-hour period to determine their proteinuria levels.

After a 12-hour fast before delivery, blood samples for vitamin D, vitamin B12, and folic acid were taken from the mother's peripheral vein; during delivery, blood samples for Netrin-1 were obtained from the umbilical vein on the umbilical cord's placental side as well as the mother's peripheral vein.

The Netrin-1 test samples were centrifuged at 3500 rpm after being allowed to stand at room temperature for 15 minutes. The resulting serum samples were kept at -80 degrees Celsius until an analysis of the Netrin-1 level was carried out. Before analysis, all kits and samples were brought to room temperature. A commercial kit from SunRed Biotechnology (Shanghai, China) was used to test samples for Human Netrin-1 (E1277Hu). Following the preparation of the standards and chemicals for the study's kits, the standards and samples were pipetted into the microplate wells. The samples were colored according to the test concentrations, as described in the prospectus. Using a Biotek Elx800 Microplate reader (BioTek Instruments Inc., Winooski, VT, USA), the absorbance values of the wells were measured at 450 nm after the color formation was observed. Serum absorbance values and the Gen5 data analysis software (Agilent Technologies, Inc., Santa Clara, CA, USA) were used to calculate concentrations.

Statistical analysis

Before starting the current investigation, we conducted a power analysis. We discovered that 95% confidence and 80% power would be obtained with at least 44 participants; at least 22 in the PE group and at least 22 in the control group. As a result, 44 people were enlisted in the study. IBM SPSS Statistics for Windows, version 25.0 (IBM Corp., Armonk, NY, USA) was used for the statistical analysis. The mean (standard deviation) was utilized for normally distributed continuous data, and the median (interquartile range) was used for data without a normal distribution. The normal distribution was evaluated using the Shapiro-Wilk test. To identify significant differences between two groups, the Mann-Whitney U test or the Student's t-test was employed. The Spearman test was used to determine correlation coefficients and the significance of relationships between ordinal variables (assuming that at least one of them has an anomalous distribution). Significance was assessed using a p-value of less than 0.05.

## Results

Maternal and umbilical cord serum Netrin-1 levels, as well as baseline characteristics of mothers and newborns

Table 1 shows that maternal age, gestational week, height, weight, BMI, and number of abortions did not differ statistically significantly between the groups (p = 0.741, p = 0.695, p = 0.653, p = 0.712, p = 0.584, and p = 0.082, respectively). The PE group's systolic and diastolic blood pressure readings were noticeably greater than those of the control group (p = 0.001 and p = 0.001, respectively). Daily proteinuria levels were significantly higher in the PE group (p = 0.001). The PE group's newborn birth weights were significantly lower than those of the controls (p = 0.036).

**Table 1:** Demographic, clinical and biochemical characteristics of the participants Data are presented as mean ± SD; Mann Whitney U test was used for statistical analysis; * p < 0.05 is considered statistically significant SD, standard deviation; BMI, body mass index; g, gram; kg, kilogram; m, meter; PE, preeclampsia

Parameters			PE (n = 22)	Control (n = 22)	p value
Maternal age, year			29.3±7.6	29.9 ±5.9	0.741 (U=213)
Gestational age, week			35.5±0.8	35.3±0.7	0.695 (U=219)
Maternal		Height, m	1.6±0.5	1.6±0.6	0.653 (U=221)
		Weight, kg	84.0±17.1	81.9±20.1	0.712 (U=215)
		BMI, kg/m^2^	32.2±6.7	31.1±7.4	0.584 (U=204)
		Parity	2.14±1.3	2.6±1.4	0.235 (U=189)
		Abortion	0.1±0.4	0.2±0.5	0.082 (U=198)
Systolic blood pressure, mmHg			182.9±15.3	115.0±14.1	0.001* (U=12)
Diastolic blood pressure, mmHg			92.3±9.2	70.9±7.5	0.001* (U=14)
Proteinuria mg/day			792.9±187.2	180.3±41.7	0.001* (U=0.00)
25-hydroxyvitamin D, ug/L			11.7±8.8	12.9±9.5	0.632 (U=215)
Vitamin B12, ug/L			214.7±92.5	197.7±83.6	0.528 (U=211)
Folic acid, ng/L			12.7±6.4	11.3±6.3	0.476 (U=215)
Serum Netrin-1, pg/ml	Maternal		867.4±192.4	702.5±186.2	0.006* (U=124)
	Umbilical cord		769.6±186.2	813.1±174.0	0.428 (U=186)
Newborn birth weight, g			2445.2±443.9	2694.8±305.6	0.036* (U=158)

Furthermore, serum levels of folic acid, vitamin B12, and vitamin D did not differ statistically significantly between the groups, as indicated in Table 1 (p = 0.632, p = 0.528, and p = 0.476, respectively). As compared to the control group, the PE group's maternal serum Netrin-1 concentrations were significantly higher (p = 0.006). Netrin-1 levels in umbilical cord blood, on the other hand, did not significantly change between groups (p = 0.428).

Correlation analyses

As shown in Table 2, Spearman correlation analysis is used to assess the associations between maternal and umbilical cord biochemical parameters and gestational age, newborn birth weight, maternal age, and BMI in the PE and control groups. Maternal age, BMI, gestational age, and newborn birth weight did not significantly correlate with maternal serum Netrin-1 levels in either the PE group (r = -0.700, p = 0. 758; r = -0.186, p = 0. 408; r = -0.215, p = 0. 336; and r = -0.186, p = 0. 158, respectively) or the control group (r = -0.383, p = 0. 078; r = 0.295, p = 0. 182; r = 0.096, p = 0. 672; and r = 0.151, p = 0.501, respectively). The correlation coefficients revealed weak associations between maternal Netrin-1 concentrations and obstetric and maternal demographic characteristics. In the PE group, there was no significant correlation between umbilical cord Netrin-1 levels and maternal age, BMI, gestational age, or newborn birth weight (r = 0.139, p = 0. 539; r = 0.219, p = 0. 329; r = 0.358, p = 0. 102; and r = 0.199, p = 0. 375, respectively). However, umbilical cord Netrin-1 levels in the control group had a significant positive correlation with both newborn birth weight (r = 0.528, p = 0.012) and gestational age (r = 0.443, p = 0.039), indicating a link between fetal Netrin-1 levels and fetal growth and maturation in normotensive pregnancies. Serum vitamin B12 levels had no significant correlation with birth weight of the newborn, gestational age, maternal age, or BMI in either group (all p>0.05). Similarly, there was no significant correlation between serum folic acid and vitamin D levels and any of the clinical parameters tested in either the PE or control groups (all p>0.05).

**Table 2:** Serum Netrin-1, vitamin B12, folic acid, and vitamin D levels correlate with maternal and fetal characteristics Spearman’s correlation analyses were used to discover the association between variables; * p < 0.05 is considered statistically significant BMI: body mass index; BW: birth weight; PE: preeclampsia

Parameters			Maternal age		Maternal BMI		Gestational age		Newborn BW	
			PE	Control	PE	Control	PE	Control	PE	Control
Serum Netrin-1 level, pg/ml	Maternal	r	-0.70	-0.383	-0.186	0.295	-0.215	0.096	-0.186	0.151
		p	0.758	0.078	0.408	0.182	0.336	0.672	0.158	0.501
	Umbilical cord	r	0.139	-0.178	0.219	0.088	0.358	0.443	0.199	0.528
		p	0.539	0.427	0.329	0.699	0.102	0.039*	0.375	0.012*
Vitamin B12, ug/L		r	0.271	0.053	0.284	0.309	-0.158	0.012	-0.228	0.178
		p	0.223	0.816	0.201	0.162	0.483	0.956	0.307	0.428
Folic acid, ng/L		r	-0.096	0.390	0.091	0.108	-0.124	0.052	-0.234	0.183
		p	0.670	0.073	0.686	0.632	0.582	0.818	0.294	0.415
25-hydroxyvitamin D, ug/L		r	-0.184	-0.042	-0.205	-0.295	0.232	-0.001	-0.183	-0.061
		p	0.412	0.853	0.360	0.182	0.298	0.998	0.415	0.787

The associations between maternal and umbilical cord biochemical parameters and clinical severity indicators like systolic and diastolic blood pressures, as well as proteinuria in the PE and control groups, were investigated using Spearman correlation analysis, as Table 3 illustrates. Maternal serum Netrin-1 levels did not significantly correlate with systolic or diastolic blood pressure in the PE group (r = 0.138, p = 0.514 and r = -0.163, p = 0.470, respectively). However, maternal serum Netrin-1 levels and proteinuria were strongly positively correlated in the PE group (r = 0.982, p = 0.001). Maternal Netrin-1 did not significantly correlate with either proteinuria or systolic or diastolic blood pressure in the control group (r = -0.152, p = 0. 499; r = -0.169, p = 0. 452; and r = -0.152, p = 0. 500, respectively). Systolic or diastolic blood pressure and umbilical cord serum Netrin-1 did not significantly correlate in either the PE (r = -0.388, p = 0.074, and r = 0.023, p = 0. 918, respectively) or control groups (r = 0.124, p = 0. 584, and r = 0.059, p = 0. 794, respectively). Furthermore, there was a significant negative correlation between umbilical cord Netrin-1 levels and proteinuria in the PE group (r = -0.560, p = 0.007), while there was no significant correlation in the control group (r = 0.091, p = 0.687).

**Table 3:** Serum Netrin-1, vitamin B12, folic acid, and vitamin D levels were correlated with systolic blood pressure, diastolic blood pressure, and proteinuria Spearman’s correlation analyses were used to discover the association between variables; * p <0.05 is considered statistically significant DBP: diastolic blood pressure; SBP: systolic blood pressure; PE: preeclampsia

Parameters			SBP		DBP		Proteinuria	
			PE	Control	PE	Control	PE	Control
Serum Netrin-1 level, pg/ml	Maternal	r	0.138	-0.152	-0.163	-0.169	0.982	0.152
		p	0.514	0.499	0.470	0.452	0.001*	0.500
	Umbilical cord	r	-0.388	0.124	0.023	0.059	-0.560	0.091
		p	0.074	0.584	0.918	0.794	0.007*	0.687
Vitamin B12, ug/L		r	-0.226	-0.335	-0.092	-0.044	0.236	0.104
		p	0.312	0.127	0.684	0.845	0.290	0.645
Folic acid, ng/L		r	-0.413	-0.018	-0.238	0.148	0.097	0.084
		p	0.056	0.936	0.287	0.510	0.668	0.710
25-hydroxyvitamin D, ug/L		r	-0.393	-0.021	-0.163	0.262	-0.51	-0.077
		p	0.070	0.926	0.470	0.239	0.820	0.734

Additionally, serum levels of vitamin D, folic acid, and vitamin B12 did not significantly correlate with systolic or diastolic blood pressure or proteinuria in either the PE or control groups (all p values > 0.05). However, there was no statistically significant correlation between systolic blood pressure and folic acid or vitamin D levels in the PE group (r = -0.413, p = 0.056 and r = -0.393, p = 0.070, respectively).

The relationships between maternal and umbilical cord serum Netrin-1 levels and serum concentrations of vitamin B12, folic acid, and vitamin D in the PE and control groups were examined using Spearman's correlation analysis, as indicated in Table 4. There were no significant correlations between maternal serum Netrin-1 levels and vitamin B12, folic acid, or vitamin D concentrations in maternal samples in either the PE group (r = 0.231, p = 0.301; r = 0.136, p = 0.547; and r = 0.031, p = 0.893, respectively) or the control group (r = -0.082, p = 0.717; r = -0.301, p = 0.174; and r = -0.246, p = 0.271, respectively). Similarly, there was no discernible association between umbilical cord serum Netrin-1 levels and vitamin B12, folic acid, or vitamin D concentrations in the PE (r = 0.220, p = 0.310; r = -0.013, p = 0.954; and r = 0.062, p = 0.784, respectively) or control groups (r = -0.187, p = 0.405; r = 0.153, p = 0.495; and r = 0.007, p = 0.974, respectively). There was no discernible association between vitamin B12 and vitamin D levels in either the PE (r = -0.075, p = 0.739) or control groups (r = -0.045, p = 0.844). In contrast, serum folic acid levels had a significant positive correlation with Vitamin D levels in both groups, with the PE group having a stronger association (r = 0.616, p = 0.002) than the control group (r = 0.511, p = 0.015).

**Table 4:** Correlation analysis between serum Netrin-1, vitamin B12, folic acid, and vitamin D levels Spearman’s correlation analyses were used to discover the association between variables; * p <0.05 is considered statistically significant PE: preeclampsia

Parameters			Vitamin B12		Folic acid		25-hydroxyvitamin D	
			PE	Control	PE	Control	PE	Control
Serum Netrin-1 level, pg/ml	Maternal	r	0.231	-0.082	0.136	-0.301	0.031	-0.246
		p	0.301	0.717	0.547	0.174	0.893	0.271
	Umbilical cord	r	0.220	-0.187	-0.013	0.153	0,062	0.007
		p	0.310	0.405	0.954	0.495	0.784	0.974
Vitamin B12, ug/L		r	-	-	0.351	0.189	-0.075	-0.045
		p	-	-	0.109	0.401	0.739	0.844
Folic Acid, ng/L		r	0.351	0.189	-	-	0.616	0.511
		p	0.109	0.401	-	-	0.002*	0.015*

## Discussion

The purpose of this study was to look into the maternal and umbilical cord levels of Netrin-1, which plays an important role in angiogenesis, endothelial/vascular smooth muscle cell morphogenesis, immune response, and inflammation in PE and control groups, as well as their relationship with vitamin D, vitamin B12, and folic acid levels. The primary finding was that the PE group had significantly higher maternal serum levels of Netrin-1. Maternal Netrin-1 levels and proteinuria were found to be significantly positively correlated in the PE group. Furthermore, we discovered that in the control group, umbilical cord Netrin-1 levels were significantly positively correlated with both gestational age and newborn weight.

Netrin-1 has been shown to be a multifunctional protein involved in angiogenesis, migration, adhesion, and cell polarity, among other cellular processes [16]. Netrins are also found in the placenta and are essential for the growth of cytotrophoblasts and the development of the placental vascular structure [17]. A study of Netrin-1 levels in pregnant women with and without PE found that mothers with PE had higher levels [18]. Furthermore, when PE was classified as mild or severe, Netrin-1 levels were discovered to be higher in severe cases [18]. A study was conducted to investigate the use of Netrin-1 as a biomarker in the diagnosis of early-onset PE because it is known to be essential for angiogenesis, which includes branching, cytotrophoblast proliferation, and placentation [19]. According to the study, early-onset PE had higher Netrin-1 levels than late-onset PE [19]. Preeclamptic pregnant women and healthy pregnant women were compared in another study. Pregnant women with severe PE were separated into mild and severe PE in order to assess Netrin-1 levels within the PE group. The study found that patients with PE had significantly higher levels of Netrin-1, with severe PE having higher levels than mild PE [20].

In our study, we found significantly higher levels of Netrin-1 in maternal serum from PE patients than in normotensive controls. Our findings are consistent with previous research demonstrating that Netrin-1 is upregulated in PE-associated conditions such as endothelial damage, hypoxia, oxidative stress, and systemic inflammation [21]. PE is primarily the result of placental hypoxia and ischemia-reperfusion injury. Hypoxia-inducible factor-1α (HIF-1α) causes endothelial and epithelial cells to express Netrin-1, which is highly activated in preeclamptic placentas [21]. The increased maternal Netrin-1 levels observed in this study may be the result of a hypoxia-driven compensatory response to preserve endothelial integrity and stop leukocyte infiltration [21].

In our study, unlike maternal findings, there was no significant difference in umbilical cord Netrin-1 levels between the preeclamptic and control groups. This finding suggests that fetal exposure to Netrin-1 persists despite significant maternal disease. This protection may be due to placental regulatory mechanisms that protect the fetus from maternal inflammatory and vascular disorders. However, in the control group, there was a significant positive relationship between umbilical cord Netrin-1 and gestational age. This finding appears biologically plausible, given that Netrin-1 is required for vascular patterning, angiogenesis, and tissue maturation during fetal development [22].

Another factor that influences PE is vitamin D, which has positive effects on placental implantation, the immune system, and angiogenic factors, as well as endothelial status and immunomodulating properties [23]. Vitamin D deficiency during pregnancy has been linked to a higher risk of PE. PE may be caused by a vitamin D deficiency, as some research indicates that pregnant women who experience PE have much lower vitamin D levels [24]. In this study, we found no statistically significant difference in vitamin D levels between the PE and control groups; however, vitamin D levels were low in both groups [24]. The PE group in our study had low vitamin D levels, which is consistent with previous research. Nevertheless, there was no discernible association between the two groups because the control group also had low vitamin D levels. This situation could be explained by the small number of participants in both groups, insufficient sun exposure, and a lack of vitamin D supplementation. Furthermore, we found no significant correlation between Netrin-1 and 25-hydroxyvitamin D levels. Although vitamin D has been shown to have immunomodulatory and endothelial effects, new research suggests that VEGF and placental growth factor, rather than Netrin-1, are the primary mediators of vitamin D's effect on angiogenic pathways.

Low maternal folate and homocysteine levels can cause perinatal complications such as neural tube defects, prematurity, low birth weight, PE, maternal and infant obesity, insulin resistance, and poor neurocognitive development [13]. Vitamin B12 and folate regulate the metabolism of homocysteine. According to the study's findings, endothelial dysfunction caused by oxidative stress in PE could be caused by hyperhomocysteinemia [13]. Homocysteine requires folate and vitamin B12 to function in enzymes such as methionine synthase. Low folate and vitamin B12 levels cause high blood homocysteine levels, which increase the risk of PE [13,14]. A study on the relationship between PE and maternal folate, homocysteine, and vitamin B12 levels discovered that high homocysteine levels increased the risk of PE, whereas high folate levels decreased it [25]. However, the same study did not find a conclusive link between PE and vitamin B12 levels [25]. Our study's findings showed that the PE and control groups' levels of folic acid and vitamin B12 did not differ significantly. Furthermore, in our study, we found no significant relationship between Netrin-1 levels and vitamin B12 or folic acid levels. Although endothelial dysfunction and poor pregnancy outcomes are known to be associated with deficiencies in micronutrients like folic acid and vitamin B12, there is currently no proof that these vitamins directly regulate Netrin-1 signaling pathways.

In our study, maternal Netrin-1 levels did not correlate with blood pressure measurements, despite the fact that the PE group had significantly higher systolic and diastolic blood pressures. This finding may be consistent with recent research indicating that the degree of endothelial dysfunction in PE is not entirely determined by hypertension [26]. A study found that Netrin-1 indirectly modulates vascular tone via nitric oxide signaling and endothelial cell survival, rather than directly affecting systemic blood pressure [27]. The idea that Netrin-1 is more closely linked to microvascular and inflammatory damage than to hemodynamic changes alone is thus supported by the lack of a correlation between Netrin-1 and blood pressure.

One of our study's most important clinical findings was a strong positive correlation between maternal serum Netrin-1 levels and proteinuria in PE. Proteinuria indicates glomerular endotheliosis and renal microvascular damage, both of which are indicative of severe disease. According to experimental and clinical studies, netrin-1 expression in renal tissue increases significantly during ischemic and inflammatory damage. It also acts as a renoprotective and anti-inflammatory mediator by reducing tubular apoptosis and leukocyte infiltration [18,28]. The strong correlation found in our study suggests that maternal Netrin-1 may serve as a biochemical indicator of renal endothelial involvement, increasing its potential utility as a marker of the disease's presence and severity.

In our study, maternal and umbilical cord Netrin-1 levels did not significantly correlate with maternal BMI, age, or number of births. While obesity and chronic low-grade inflammation are associated, new research suggests that Netrin-1 dysregulation is more strongly associated with vascular inflammation than with obesity itself, particularly in pregnancy-related conditions [29]. The idea that the Netrin-1 alterations seen in this study are disease-specific rather than brought on by baseline maternal characteristics is supported by the lack of correlation with these demographic factors [29].

The current study's limitations were as follows: it was a single-center study; it had a small sample size; netrins other than Netrin-1 were not examined due to financial constraints; a mild PE group was not established; and only pregnancies at 34-36 weeks of gestation were assessed.

## Conclusions

We found some important findings in this study when we compared PE, Netrin-1, vitamin D, and folic acid levels, as well as these levels with each other and with the participants' obstetric and clinical parameters. We discovered that PE cases had higher levels of maternal Netrin-1, and that maternal Netrin-1 levels and proteinuria were significantly positively correlated. Nevertheless, we did not find any correlation or increase in umbilical cord Netrin-1 levels. We believe that the primary reason for these changes is a compensatory mechanism designed to protect the fetus from endothelial and microvascular damage. More research is needed to determine this.
